# Housing and Social Environments of African (*Loxodonta africana*) and Asian (*Elephas maximus*) Elephants in North American Zoos

**DOI:** 10.1371/journal.pone.0146703

**Published:** 2016-07-14

**Authors:** Cheryl L. Meehan, Jennifer N. Hogan, Mary K. Bonaparte-Saller, Joy A. Mench

**Affiliations:** 1 AWARE Institute, Portland, Oregon, United States of America; 2 Department of Animal Science and Center for Animal Welfare, University of California Davis, Davis, California, United States of America; University of Tasmania, AUSTRALIA

## Abstract

We evaluated 255 African (*Loxodonta africana*) and Asian (*Elephas maximus*) elephants living in 68 North American zoos over one year to quantify housing and social variables. All parameters were quantified for the both the day and the night and comparisons were made across these time periods as well as by species and sex. To assess housing, we evaluated not only total exhibit size, but also individual animals’ experiences based on the time they spent in the unique environments into which the exhibits were subdivided. Variables developed to assess housing included measurements of area as a function of time (Total Space Experience), environment type (Indoor, Outdoor, In/Out Choice) and time spent on hard and soft flooring. Over the year, Total Space Experience values ranged from 1,273 square feet to 169,692 square feet, with Day values significantly greater than Night values (p<0.001). Elephants spent an average of 55.1% of their time outdoors, 28.9% indoors, and 16% in areas with a choice between being in or out. Time spent on hard flooring substrate ranged from 0% to 66.7%, with Night values significantly greater than Day (p<0.001). Social factors included number of animals functionally housed together (Social Experience) and social group characteristics such as time spent with juveniles and in mixed-sex groups. Overall Social Experience scores ranged from 1 to 11.2 and were significantly greater during the Day than at Night (p<0.001). There were few significant social or housing differences between African (N = 138) and Asian (N = 117) species or between males (N = 54) and females (N = 201). The most notable exception was Total Space Experience, with African and male elephants having larger Total Space Experience than Asian and female elephants, respectively (P-value<0.05). The housing and social variables evaluated herein have been used in a series of subsequent epidemiological analyses relating to various elephant welfare outcomes.

## Introduction

Addressing questions regarding zoo elephant welfare is important, as significant public attention has been directed toward the housing and care of elephants in zoos. Of particular interest is an enhanced understanding of elephant housing and management as they relate to welfare outcomes. In order to determine the factors most salient to elephant welfare and inform evidence-based elephant care, elephant housing and management and their associations with elephant welfare must be systematically assessed in zoological settings.

Comprehensive animal welfare assessment relies on the collection and analysis of two distinct, yet related, types of data [1]. The first describes housing features and management practices—also known as resource-based measures, and the second requires the measurement of welfare indicators such as behavior, physical health and physiology–also known as outcome-based measures. In most cases resource-based measures must be assessed in conjunction with welfare outcomes in order to understand the animals— responses to variation in environmental parameters. This can be accomplished either through experimental means by comparing welfare outcomes of animals exposed to different housing or care conditions (i.e. [2,3,4,5]) or through epidemiological studies correlating resource-based parameters and welfare outcomes within a diverse population (i.e. [6,7,8]).

Using resource-based parameters as independent variables in an epidemiological study requires the development of measurement methods that account for significant variation in form and practice within the population. This paper focuses on the development and analysis of variables relating to housing and social aspects of elephant management in accredited zoos in North America. The dual purpose was to provide a comprehensive review of elephant housing and social management in zoos and to generate appropriate independent variables to be used in subsequent epidemiological analyses of behavioral [9,10,11] physiological [12] and health-related [13,14] welfare indicators.

Our study focused on environmental and social factors because both of these play an important role in the behavior and ecology of wild elephants [15, 16, 17, 18] and because research in many managed species demonstrates that animals’ experiences of physical space and social milieu play a critical role in their welfare. For example, studies have shown that for social species, isolation, exposure to groups of unnatural size or composition, or repeated disruption of established social groups have detrimental effects on physiology behavior, and e psychological state [19, 20]. Conversely, social environments can be used to promote positive welfare in managed animals by increasing mental stimulation, promoting social learning and the expression of highly motivated and/or natural behaviors, and by buffering stress [21, 22].

The physical environment also plays an important role in the welfare of managed animals across contexts and species [23]. One key component of the physical environment is the amount of space to which an animal has access. Variation in the amount of space available to animals has been shown to affect welfare, although these effects vary. Some studies show that experimental decreases in space allowance result in negative effects such as increases in aggressive behaviors, adrenocortical secretion [3], and stereotypic behavior [4,24]. Other studies, however, have failed to demonstrate associations between smaller spaces and indicators of compromised welfare [6, 25, 26, 27]. Differences in the effect of space allowance on welfare indicators such as stereotypic behavior may be attributable to the natural history of the species such that spatial restriction plays a larger role in welfare outcomes for species with large home ranges (e.g. carnivores:[28]).

The quality of space experienced by animals is also important. For elephants, flooring and substrate composition may be particularly critical aspects of environmental quality. Hard surfaces have been associated with poor elephant foot and joint conditions including trauma to foot pads, toenails, joints and other musculoskeletal structures [29, 30, 31, 32]. Studies in cattle have shown similar trauma associated with hard surfaces [33, 34], as well as a protective effect of soft substrates [35].

To date, no studies have systematically evaluated the effects of social and housing factors on the welfare of zoo elephants, nor is there information about how species and sex contribute to variation in these factors. Given the importance of resource-based measures in developing an understanding of elephant management and facilitating welfare assessment, our study was designed to collect detailed information about the housing and social management of zoo elephants in a way that captured the variation in these factors both within zoos and across the zoo population. Subsequently, we translated these data into standardized variables suitable for descriptive and comparative analyses. A similar approach was taken in a related manuscript that to quantifies other elephant management factors for this population including enrichment, training, feeding and exercise [36].

## Materials and Methods

### Ethics Statement

This study was authorized by the management at each participating zoo and, where applicable, was reviewed and approved by zoo research committees. In addition the study protocol was reviewed and approved by the Zoological Society of San Diego Institutional Animal Care and Use Committee N.I.H. Assurance A3675-01; Protocol 11–203. The study was non-invasive.

### Data Collection

The evaluation of housing and social resource-based parameters in multi-institutional zoo studies is typically conducted using facility-level or herd-level surveys [36, 37]. However, exploratory conversations with participating elephant care professionals at the outset of this project revealed significant variation not only between zoos, but also within zoos with respect to how individual elephants were managed spatially and socially. Within zoos, elephant managers often vary housing options and social groupings to account for time of day, time of year, herd dynamics, husbandry schedules, and individual elephant characteristics. Therefore, in order to accurately measure factors related to zoo elephants’ housing and social environments across the population of elephants, we developed a data model and accompanying software interface to capture both the range in complexity between zoos and the variability at the individual elephant level within zoos.

The process for capturing these data included two integrated steps completed by each participating zoo. The first step was the zoo registration process, which captured data relating to demographics, exhibit characteristics, and social groupings (Table 1). The second step was submission of monthly Management Logs reporting housing and social time budgets for each elephant. Each zoo completed these logs for day management and for night management every month for 12 months. To account for variation and fluctuation in how day and night were defined by the zoos, the number of hours associated with day management (Day) and night management (Night) were reported by each zoo on a monthly basis. These management periods generally coincided with employee schedules (e.g., day management meant that employees were onsite), but varied within and between facilities due to seasonal and geographic differences.

**Table 1:** Data captured through the zoo registration process and web portal that were used as the basis for variable creation.

Data Category	Parameters
Demographics	
For Each Elephant	Name
	Date of Birth
	Species
	Sex
	Studbook Number
Exhibit	
Enclosures: defined as each individual unit of space available for housing elephants.	Area
	Exposure (Indoors / Outdoors / In/Out Choice)
	Flooring Substrate Types and Percent Coverages
Environments: defined as the ways that enclosures were used individually or in combination (by opening doors/gates to join adjacent areas) to house elephants	Included Enclosures
	Area*
	Exposure (Indoors / Outdoors / In/Out Choice)*
	Flooring Substrate Types and Percent Coverages*
Social Groupings	
Social Groups: defined as all groupings of elephants used in the course of normal management. Elephants considered to be in a social group must share unrestricted space.	Members of Group
	Time Period (when each group occurs): Day, Night or Both

*Calculated by software based on enclosure level data

**Fig 1:**
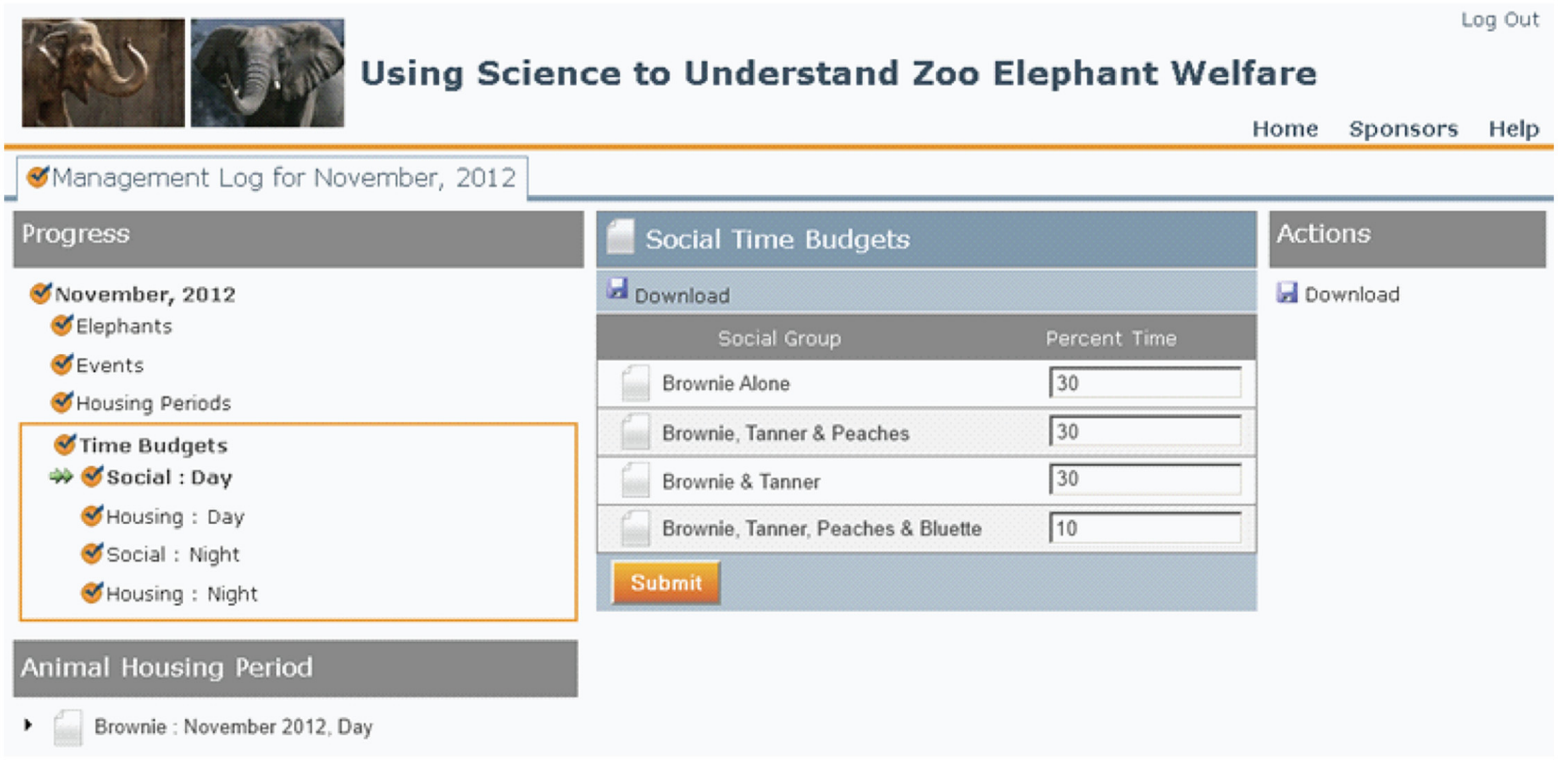
Illustration of the process by which elephant managers provided social time budget information for an elephant (Brownie) that was a member of 4 social groups and spent time in each social group option during the Day during November. This interface was presented sequentially for all elephants at a zoo. When applicable, fields were auto-filled to reflect the fact that by definition time assigned to one member of a social group must apply to all members of that social group. The software also verified that the values entered summed to 100.

Social time budgets were based on how each individual elephant’s time was spent in each of the social groups of which it was a member. Social groups were defined as being comprised of elephants that shared unrestricted space during the course of normal social management. Additionally, when elephants were reported to spend time housed alone, managers were asked to report the percentage of time housed alone that was spent with restricted access (via a barrier) to one or more other elephants. An example of the software interface for the social time budget of an elephant is provided in Fig 1.

**Fig 2:**
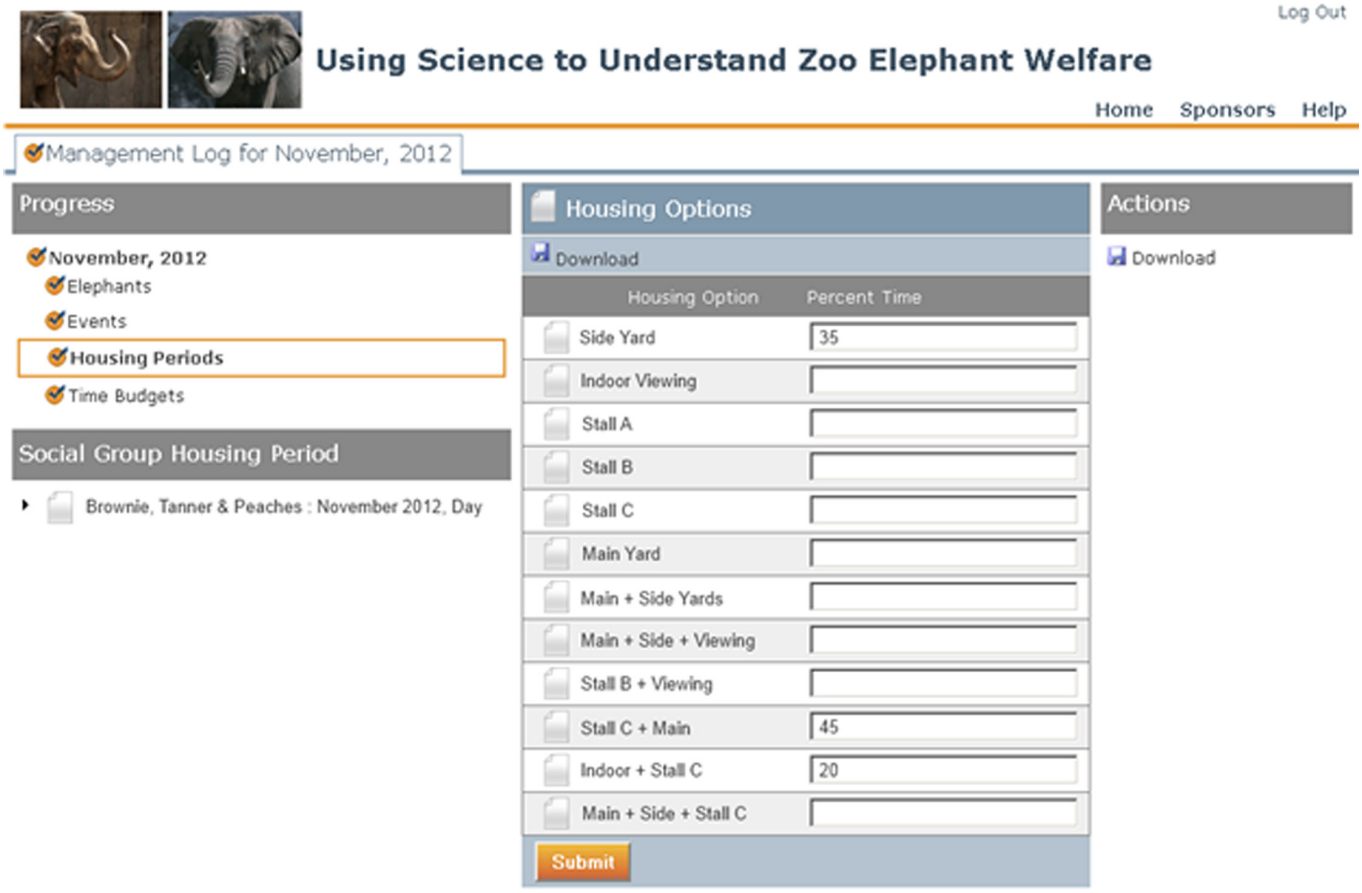
Illustration of the process by which elephant managers provided housing time budget information for the hypothetical social group “Brownie, Tanner and Peaches”, which spent time in three environments during the Day during November. This interface was presented sequentially for all social groups and the software assigned values to all members of the social group in the database. The software also verified that the values entered summed to 100.

Housing time budgets allowed zoos to report how much time each social group spent in each of the available environments. Environments were defined as single or multiple contiguous units of space in which elephants were housed during the course of normal management. An example of the software interface for the housing time budget is provided in Fig 2.

All data were stored in a relational database using SQL server. Confidentiality of data was ensured by using randomly generated unique alpha-numeric codes for zoos and elephants. Data were exported to MS Excel (Seattle, WA) and SAS 9.3 (Cary, NC) for variable creation.

### Subjects

Monthly Management Logs were completed by 68 zoos, which represented 96% of the AZA accredited elephant holding institutions in 2012. Elephant-level data were only included for analysis if the elephant was not born, did not die, and was not transferred between zoos during the period from January 1, 2012 to December 31, 2012. These criteria were met by 255 elephants, 138 of which were African (110 females and 28 males) and 117 of which were Asian (91 females and 26 males). Cases where sample sizes varied due to data availability / applicability are noted in the results.

### Variable Creation

While data were collected monthly, all results presented herein represent yearly averages. Descriptions of all calculated variables are presented in Table 2; the following paragraphs describe these variables in more detail.

**Table 2:** Description of variables created from the space and social information on manager’s survey, indicating unit of analysis, unit of measurement, time scale for which each variable was evaluated, and calculation method.

Variable	Category	Unit of Analysis	Unit*	Time Scale	Description
Total Exhibit Size		Zoo	(ft^2^)		Total area of space available to elephants at zoo
Herd Size		Zoo			Total number of elephants at zoo
Environment Count		Zoo			Total number of unique spaces into which an exhibit could be configured
Contact	Environment	Elephant		Overall, Day, Night	Maximum number of unique environments an elephant was housed in
	Animal	Elephant		Overall, Day, Night	Maximum number of unique elephants focal animal is in contact with
	Social Group	Elephant		Overall, Day, Night	Maximum number of unique social groups focal animal is part of
Space Experience					The average weighted (by percent time) size of all environments in which an elephant spent time.
	Total	Elephant	(ft^2^)	Overall, Day, Night	For all environment types
	Indoor	Elephant	(ft^2^)	Overall, Day, Night	For indoor environments only
	In/Out Choice	Elephant	(ft^2^)	Overall, Day, Night	For environments where there is a choice of indoors or outdoors
	Outdoor	Elephant	(ft^2^)	Overall, Day, Night	For outdoor environments only
Space Experience by Elephant		Elephant	(ft^2^)	Overall	The area of all environments in which an elephant spent time, divided by the number of elephants sharing each environment, weighted by the percent time spent in each environment and averaged.
Relative Space Experience Change		Elephant			(Total Day Space Experience—Total Night Space Experience)/(Total Day Space Experience)
Proportion Space Experienced		Elephant	%		Proportion of Total Overall Space Experience to Total Exhibit Size
Social Experience		Elephant		Overall, Day, Night	The average weighted (by percent time) size of all social groups in which an elephant spent time.
Relative Social Experience Change		Elephant			(Total Day Social Experience—Total Night Social Experience)/(Total Day Social Experience)
Proportion Social Experienced		Elephant	%		Proportion of Overall Social Experience to Herd Size
Percent Time					Sum of monthly percent time spent in category, averaged over time period
	Indoor	Elephant	%	Overall, Day, Night	Time spent in indoor environments
	In/Out Choice	Elephant	%	Overall, Day, Night	Time spent in environments with an indoor/outdoor choice
	Outdoor	Elephant	%	Overall, Day, Night	Time spent in outdoor environments
	Soft Substrate	Elephant	%	Overall, Day, Night	Time spent in environment with 100% grass, sand, or rubber substrate
	Hard Substrate	Elephant	%	Overall, Day, Night	Time spent in environment with 100% concrete or stone aggregate substrate
	Dirt Substrate	Elephant	%	Overall, Day, Night	Time spent in environment with 100% dirt substrate
	Juveniles (<7 years old)	Elephant	%	Overall, Day, Night	Time spent in social groups where an elephant 7 years or younger was present
	Mixed Sex Groups	Elephant	%	Overall, Day, Night	Time spent in social groups where both males and females were present
	Housed Separately	Elephant	%	Overall, Day, Night	Time spent housed alone (Social group of 1)
	Housed Separately with Restricted Physical Access	Elephant	%	Overall, Day, Night	Percentage of elephant’s time Housed Separately with contact through a barrier.

*Area based variables are presented as ft^2^ for consistency with companion papers. Metric equivalents are available in (S1 Table).

#### Social

Each elephant’s social situation was evaluated using several distinct variables. Herd Size was defined as the maximum number of elephants present at a zoo during the 2012 study period. Within a zoo, elephants were combined into social groups for management purposes and individual elephants spent varying amounts of time in the social groups of which they were members. Social groups were defined as groups of elephants that shared physical space without an intervening barrier, and each Social Group was comprised of a unique set of animals. Animal Contact was defined as the maximum number of unique elephants (not including itself) with which an elephant shared social groups and Social Group Contact was defined as the maximum number of unique social groups of which an elephant was a member per management period (Day, Night, and Overall) throughout the study.

The percent of time each elephant spent in each social group during each management period (Day, Night, and Overall) each month was taken from the monthly Management Logs. Social Experience (Eq 1) was calculated by taking the size of each social group in which an elephant spent time, multiplying it by the percentage of time the elephant spent in that social group and then averaging these weighted social group sizes. Social Experience was calculated per management period (Day, Night, and Overall). Relative Social Experience Change was calculated by taking the difference between Day Social Experience and Night Social Experience and dividing by the Day Social Experience. This relative value typically ranged from -1 to 1, with values close to zero indicating similar day and night experiences, values close to 1 indicating a larger social experience during the day, and values close to -1 indicating a larger social experience at night.

$$Social\;Experience=\frac{\sum_{i=1}^{n}\left(\left(percent\;time\;spent\;in\;social\;groups\;x_{i}\right)*\left(animal\;count\;in\;social\;group\;x_{i}\right)\right)}{\sum_{i=1}^{n}\left(percent\;time\;spent\;in\;social\;group\;x_{i}\right)}$$ (1)

Social groups were classified by the presence or absence of a juvenile (defined as 7 years of age or younger), the presence of one or both sexes, and the number of elephants in the group. Elephants housed without a social partner (Social Group size of 1) were considered to be Housed Separately. An elephant was considered Housed Separately with Restricted Physical Access if it could see and/or touch another elephant but was in a physically separated environment. Percent Time in a social group was calculated by first summing the percent time spent in that social group in a given month, then averaging the monthly sums. This calculation was performed per social group type (with Juveniles, with Mixed Sex, Housed Separately, and Housed Separately with Restricted Physical Access) and per housing period (Day, Night, and Overall).

#### Housing

Ten separate housing variables were calculated. Total Exhibit Size was defined as the total area (square feet) of space available to elephants within a zoo. Exhibits are comprised of multiple units of space (Enclosures), and Environments are the unique ways in which Enclosures are used singly or in combination to house elephants. The total number of Environments available at each zoo was tabulated as Environment Count, and the maximum number of unique environments that each elephant spent time in during the 12 month study period was calculated as Environment Contact.

Environments were classified as being indoors, outdoors, or comprised of both indoor and outdoor areas (In/Out Choice). The percent of time each elephant spent in each Environment during the Day and Night for each month was calculated by multiplying the percent time the elephant spent in a given Social Group by the percent time the Social Group spent in each Environment. Using this information, several elephant level space variables were created. Space Experience (Eq 2) was calculated by taking the size of each environment in which an elephant spent time, multiplying it by the percentage of time the elephant spent in that environment and then averaging these weighted environment sizes.

Space Experience was calculated for all environment types combined (Total), and for each of the three environment types separately (Indoor, Outdoor, and In/Out Choice). Space Experience for each of these was calculated for Day, Night and Overall. So, for example, the Night Outdoor Space Experience describes the average size of the outdoor environments the elephant spent time in at night, weighted by the amount of time spent in each outdoor environment at night. Throughout the rest of the manuscript, similar differentiations will be presented using a “per X” designation, e.g. Space Experience was calculated per environment type (Total, Indoor, Outdoor, and In/Out Choice) and per housing period (Day, Night, and Overall).

$$Space\;Experience=\frac{\sum_{i=1}^{n}\left(\left(percent\;time\;spent\;in\;environment\;x_{i}\right)*\left(environment\;x_{i}\;size\right)\right)}{\sum_{i=1}^{n}\left(percent\;time\;spent\;in\;environment\;x_{i}\right)}$$ (2)

Space Experience by Elephant was calculated much as the Total Space Experience variable was calculated, except that environment area was divided by the total number of elephants using the space at that time. This allowed for Space Experience to reflect elephant density within environments. Relative Space Experience Change was calculated by taking the difference between the Day Total Space Experience and the Night Total Space Experience and dividing by the Day Total Space Experience. This relative value typically ranged from -1 to 1, with values closer to zero indicating similar Day and Night Space Experiences, values closer to 1 indicating a larger Day Space Experience, and values closer to -1 indicating a larger Night Space Experience.

Percent Time in an environment type was calculated by first summing the percent time spent in each environment for a given month, then averaging the monthly sums. This calculation was performed for each individual environment type (Indoor, Outdoor, and In/Out Choice) and per housing period (Day, Night, and Overall).

#### Flooring

Seven classes of flooring substrate were defined: grass, sand, rubber padding, dirt, stone aggregate, concrete, and other. We categorized the types of substrates into hard surface (concrete and stone aggregate), soft surface (grass, sand, and rubber padding), and dirt. However, our data collection methods allowed us to detect the fact that many environments were comprised of multiple flooring substrates, including both hard and soft. We had information regarding the percent coverage of each type of substrate within an environment, but not the configuration. Furthermore, we also did not have information regarding what portion of the environments were used by an elephant, just that an elephant had access to the substrate. We were therefore not able to determine either the contiguous coverage area or the time elephants spent on each of the different substrate types in mixed-substrate environments. We therefore focused the analysis on substrate categories where we knew the environment consisted of 100% coverage of hard substrate, 100% coverage of soft substrate, or 100% coverage of dirt. This is a conservative approach, as time spent in environments with substrate coverage that was large, but less than 100%, was not captured in this analysis. Environments with 100% coverage of dirt substrate were classified as a separate category because we were informed that dirt can be either soft or hard depending on how it is managed; however, we had not collected sufficient information to make this distinction within our dataset. Percent Time on a substrate was calculated by first summing the percent time spent in environments with 100% coverage of that substrate for a given month, then averaging the monthly sums. This calculation was performed per substrate type (Soft, Hard, and Dirt) and per housing period (Day, Night, and Overall).

### Statistical Analyses

Descriptive statistics, including the range, mean, and standard deviation, were calculated for all variables. Most variables were determined to be non-normally distributed, so non-parametric tests were used for population comparisons. Matched pairs for day and night housing were compared using the Wilcoxon Signed Rank Test. The variables were also assessed for species and sex differences. Means and standard deviations were calculated for variables and the Mann-Whitney U (Wilcoxon Rank Sum) Test was used to determine differences attributable to species or sex. Total Exhibit Space and Total Overall Space Experience were compared using the Wilcoxon Signed Rank Test for matched pairs. Proportion Space Experienced was determined by comparing the proportion of an elephant’s Space Experience to the Total Exhibit Size, where 100% would indicate that an elephant’s Space Experience matched the Total Exhibit Size at that zoo. Herd Size and Overall Social Experience were compared using the Wilcoxon Signed Rank Test for matched pairs. Proportion Social Experienced was calculated as the proportion of Social Experience to Herd Size. Statistical analyses were conducted using SAS software, version 9.3 (SAS Institute, Inc., Cary, NC), and a *P-*value of <0.05 was considered statistically significant.

## Results

**Fig 3:**
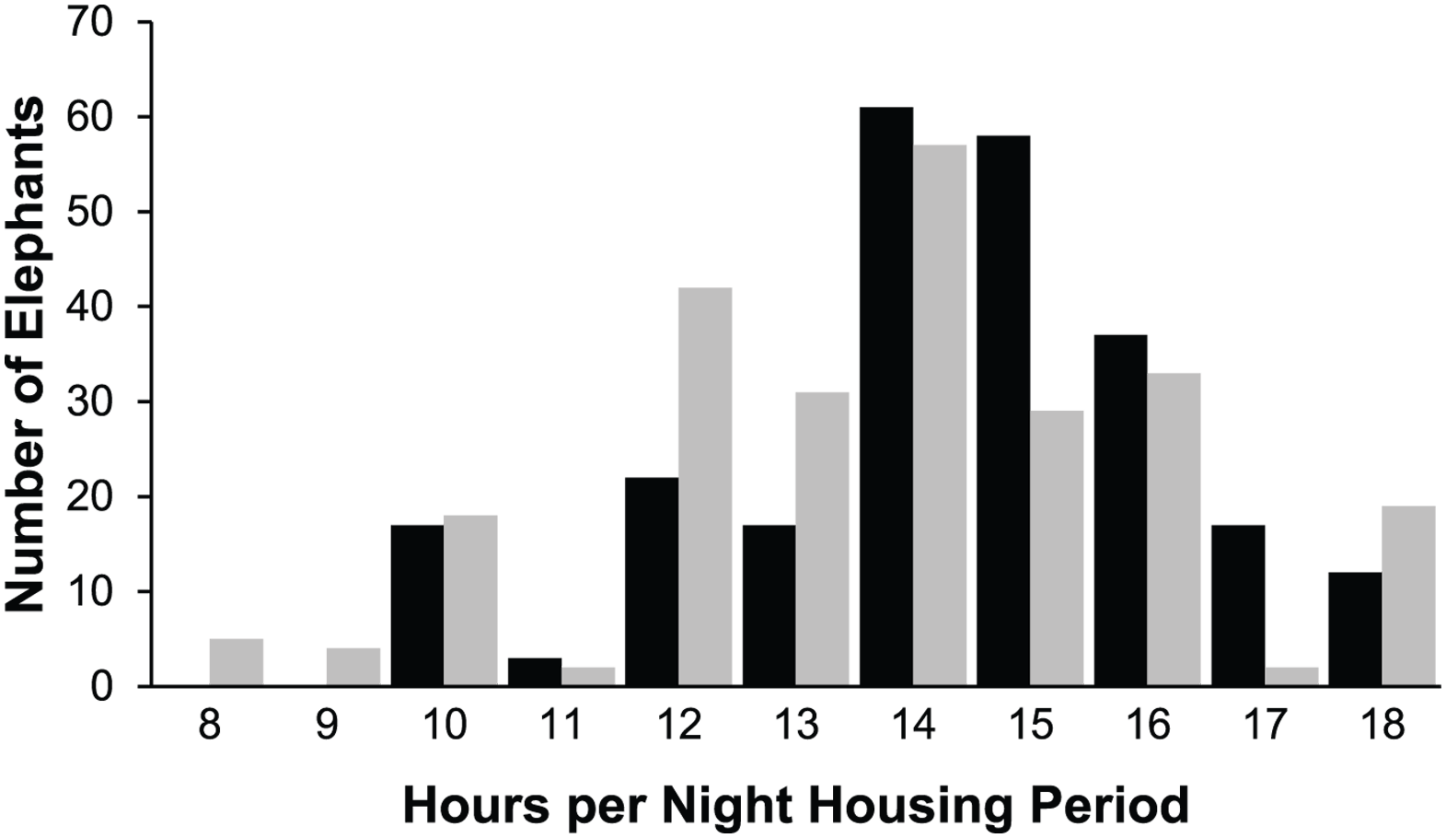
The number of elephants with various Night lengths. Black bars indicate January, grey bars indicate July.

Twenty-three housing and social variables were created (Table 2). As many variables use hours per day and night management periods as a basis for calculation, the population range for night lengths and distribution of elephants with respect to night length are shown in Fig 3. Night length varied monthly, with an average across all months of 12.5 hours. Data for one winter (January) and one summer (July) month are presented for comparison. The modal Night length for both January and July was 14 hours, with a range among all elephants of 10–18 hours in January and 8–18 hours in July.

**Fig 4:**
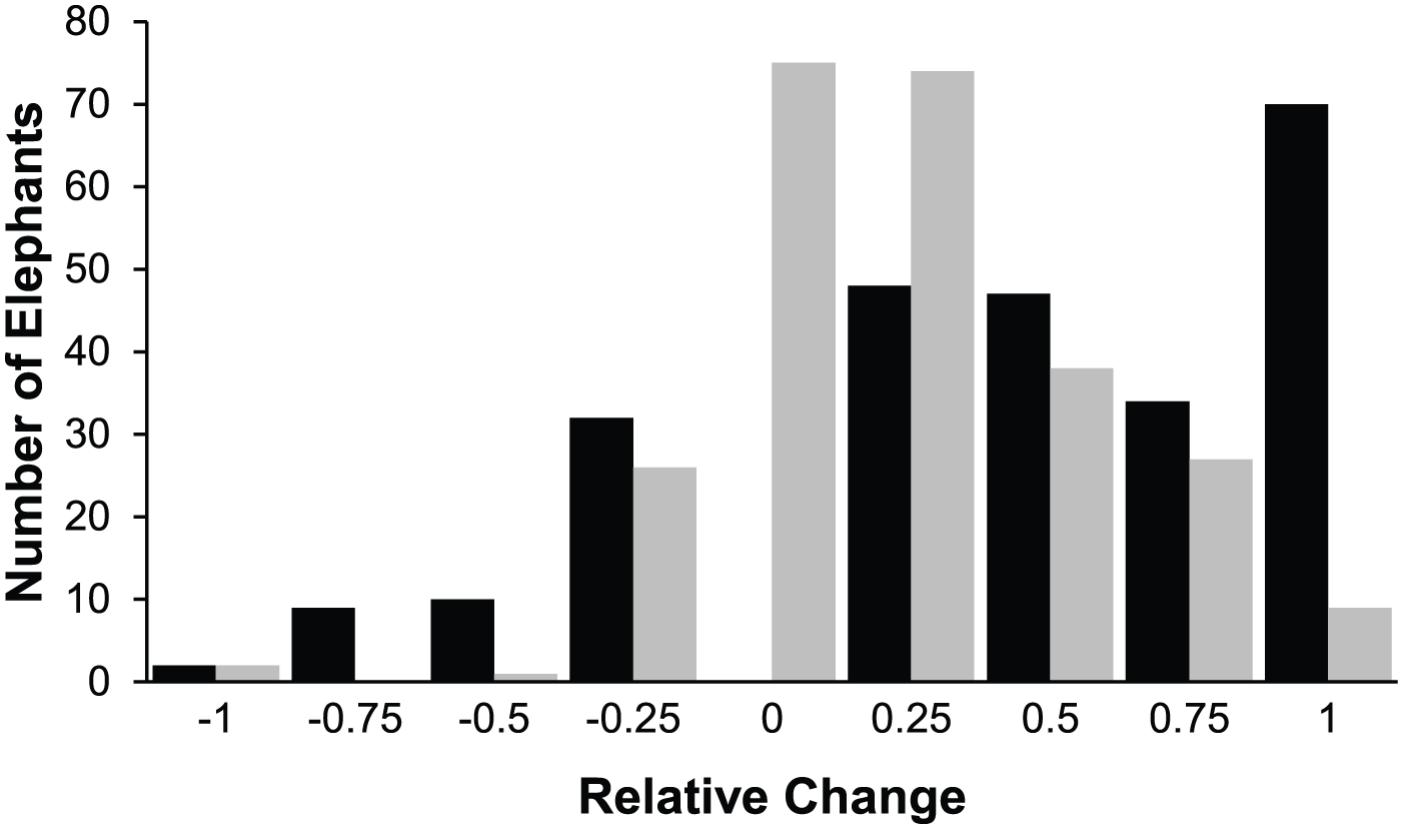
The number of elephants with particular amounts of relative space and social change from Day to Night. Black bars indicate relative space experience change and grey bars indicate relative social experience change. Values close to zero indicate no day to night change, values close to 1 indicate greater experience in the day and values close to -1 indicate larger experience at night.

Table 3 lists the arithmetic mean, standard error, and range for each variable for Day, Night, and Overall management periods. Comparing the elephants’ Day and Night experiences revealed several significant differences. Day values were greater for both Total Space Experience (Day = 38, 980.4 ft^2^; Night = 22,098.3 ft^2^) and Outdoor Space Experience (Day = 44,515.2 ft^2^; Night = 26,636.4 ft^2^). However, Indoor Space Experience was greater during the Night, and elephants spent a higher percentage of their time indoors at Night than during the Day (43.4% compared to 14.3%). Social Experience was greater during the Day (3.1) than at Night (2.4), and elephants spent more time at Night (35.1%) housed separately than during the Day (18.3%). The relative space and social experience change between Night and Day is shown in Fig 4. For most elephants, both space and social experiences were greater during the Day than at Night.

**Table 3:** Housing, flooring, and social variables for the population (Overall, Day, Night) showing means, standard errors, and ranges. Day and Night values were compared using the Wilcoxon Signed Rank Test.

		Overall				Day				Night				Day to Night Comparison	
Variable	N	Mean	SEM	Min	Max	Mean	SEM	Min	Max	Mean	SEM	Min	Max	*P*-value	
*Housing*															
Total Exhibit Size (ft^2^)	68	76767.1	5872.1	7728.5	347007.7	-	-	-	-	-	-	-	-	-	
Environment Count	68	12.5	1.2	2.0	62.0	-	-	-	-	-	-	-	-	-	
Environment Contact	252	9.4	0.4	2.0	46.0	6.7	0.3	1.0	35.0	6.2	0.3	1.0	33.0	0.054	
Space Experience Total (ft^2^)	252	30253.0	2003.4	1273.4	169691.8	38980.4	2446.6	1184.0	159031.2	22098.3	1981.5	444.6	209573.1	<0.001	*
Space Experience by elephant (ft^2^)	252	11136.3	731.5	328.5	100842.9	13749.8	838.1	375.7	86257.6	8771.0	738.6	242.2	120820.5	<0.001	*
Relative Space Experience Change	252	0.37	0.03	-1.20	0.99	-	-	-	-	-	-	-	-	-	
Proportion Space Experienced (%)	252	34.8	1.3	5.3	96.1	-	-	-	-	-	-	-			
Space Experience Indoor (ft^2^)	252	1393.9	89.3	0.0	8603.6	1176.5	92.2	0.0	9767.2	1391.8	84.1	0.0	7288.2	0.001	*
Space Experience In/Out Choice (ft^2^)	252	16089.9	1730.2	0.0	156374.7	8778.0	1,215.5	0.0	135880.2	16015.6	1800.3	0.0	181067.1	<0.001	*
Space Experience Outdoor (ft^2^)	252	42203.1	2429.8	790.1	194368.1	44515.2	2,592.2	790.1	179026.3	26636.4	2554.4	0.0	287140.0	<0.001	*
Time Indoor (%)	252	28.9	1.4	0.0	81.0	14.3	1.2	0.0	79.0	43.4	2.1	0.0	100.0	<0.001	*
Time In/Out Choice (%)	252	16.0	1.3	0.0	89.8	8.5	1.1	0.0	89.6	23.4	1.9	0.0	100.0	<0.001	*
Time Outdoor (%)	252	55.1	1.6	1.3	100.0	77.1	1.6	2.7	100.0	33.2	2.1	0.0	100.0	<0.001	*
*Flooring*															
Time on Soft Substrate (%)	252	10.6	0.9	0.0	58.3	6.6	0.7	0.0	38.8	11.0	1.1	0.0	66.7	<0.001	*
Time on Hard Substrate (%)	252	10.2	0.8	0.0	66.7	4.8	0.5	0.0	27.1	15.0	1.3	0.0	66.7	<0.001	*
Time on Dirt Substrate (%)	252	0.8	0.3	0.0	43.9	0.7	0.3	0.0	40.3	0.9	0.4	0.0	50.7	0.0352	*
*Social*															
Herd Size	68	3.8	0.1	1.0	13.0	-	-	-	-	-	-	-	-	-	
Animal Contact	252	2.7	0.2	0.0	11.0	2.7	0.2	0.0	11.0	1.9	0.2	0.0	11.0	<0.001	*
Social Group Contact	252	3.9	0.3	1.0	30.0	3.1	0.2	1.0	18.0	2.5	0.2	1.0	21.0	<0.001	*
Social Experience	252	2.7	0.1	1.0	11.2	3.1	0.2	1.0	12.7	2.4	0.1	1.0	10.6	<0.001	*
Relative Social Experience Change	252	0.16	0.02	-1.04	0.90	-	-	-	-	-	-	-	-	-	
Proportion Social Experienced (%)	252	60.0	1.7	5.3	100.0	-	-	-	-	-	-	-			
Time with Juveniles (<7 years old) (%)	252	21.1	2.3	0.0	100.0	22.6	2.4	0.0	100.0	19.7	2.3	0.0	100.0	0.178	
Time with Mixed Sex Groups (%)	252	20.3	2.2	0.0	100.0	23.8	1.5	0.0	100.0	16.9	2.2	0.0	100.0	<0.001	*
Time Housed Separately (%)	252	26.6	2.2	0.0	100.0	18.3	1.2	0.0	100.0	35.1	2.7	0.0	100.0	<0.001	*
Time Housed Separately with Restricted Physical Access (%)	71	37.2	3.0	0.0	100.0	24.2	2.0	0.0	100.0	52.3	2.6	0.0	100.0	<0.001	*

* Indicates *P*-value<0.05

Of the 68 zoos assessed in this study, 45.5% (31/68) of zoos housed Asian elephants only, and 48.5% (33/68) housed African elephants only. Four zoos housed both African and Asian elephants. With respect to sex, 54.4% (36/68) of zoos housed female elephants only, and 45.5% (31/68) of zoos housed both males and females. Only one zoo housed male elephants only, and had three males. As seen in Table 4, the vast majority of measures did not differ due to either sex or species: only four of the 23 measures differed significantly between African and Asian elephants and only three of the 23 measures differed significantly between males and females. In terms of species differences, Africans had a larger Total Space Experience and spent time in more unique environments (Environment Contact) than Asians. Similarly, Overall Outdoor Space Experience was significantly greater for African elephants: African elephants experienced 52,703.3 ft^2^ compared to the Asian elephant experience of 29,382.2 ft^2^.

**Table 4:** Housing, flooring, and social variables (Overall) by species and sex including means and standard errors. Comparisons between African and Asian elephants, and male and female elephants, were made using the Mann-Whitney U (Wilcoxon Rank Sum) test.

	Species								Sex							
	African			Asian					Male			Female				
Variable	N	Mean	SEM	N	Mean	SEM	*P*-value		N	Mean	SEM	N	Mean	SEM	*P*-value	
*Housing*																
Total Exhibit Size (ft^2^)	136	113,648.5	8,105.9	114	97,341.2	8,467.6	0.042	*	52	115,547.2	14,352.6	198	103,760.8	6,393.6	0.663	
Environment Contact	135	9.9	0.5	117	8.9	0.7	0.009	*	54	10.0	0.8	198	9.2	0.5	0.098	
Space Experience (ft^2^)	135	39,338.8	3,345.1	117	19,264.2	1,327.1	<0.001	*	54	40,584.2	5,636.6	198	27,136.9	1,980.4	0.027	*
Space Experience by Elephant (ft^2^)	135	12,374.5	1,192.4	117	9,707.5	752.3	0.162		54	18,848.3	2,638.2	198	9,033.1	502.3	0.162	
Relative Space Experience Change	135	0.40	0.04	115	0.33	0.04	0.361		54	0.36	0.06	198	0.37	0.03	0.630	
Proportion Space Experienced (%)																
Space Experience Indoor (ft^2^)	135	1,285.2	101.1	117	1,500.5	138.7	0.518		54	1,520.9	183.4	198	1,347.6	94.9	0.436	
Space Experience In/Out Choice (ft^2^)	135	19,113.5	2,727.5	117	12,602.4	1,959.7	0.726		54	25,330.7	5,332.1	198	13,570.1	1,618.0	0.042	*
Space Experience Outdoor (ft^2^)	135	52,703.3	3,887.7	117	29,382.2	2,160.9	<0.001	*	54	52,293.2	6,590.5	198	3,9034.2	2,475.9	0.092	
Time Indoor (%)	135	27.0	2.0	117	31.1	2.1	0.162		54	24.3	3.1	198	30.2	1.6	0.079	
Time In/Out Choice (%)	135	16.0	1.7	117	15.9	2.1	0.620		54	17.4	2.8	198	15.6	1.5	0.202	
Time Outdoor (%)	135	57.0	2.2	117	53.0	2.4	0.206		54	58.2	3.4	198	54.3	1.8	0.325	
*Flooring*																
Time on Soft Substrate (%)	135	10.4	1.6	117	10.8	1.1	0.306		54	12.0	1.9	198	10.2	1.0	0.375	
Time on Hard Substrate (%)	135	10.6	1.1	117	9.7	1.2	0.253		54	8.1	1.5	198	10.7	1.0	0.189	
Time on Dirt (%)	135	1.4	0.6	117	0.2	0.1	0.085		54	1.2	0.9	198	0.8	0.3	0.507	
*Social*																
Herd Size	136	5.3	0.3	116	4.9	0.3	0.195		54	4.9	0.4	198	5.2	0.2	0.582	
Animal Contact	136	2.7	0.2	116	2.7	0.2	0.734		54	2.4	0.4	198	2.8	0.2	0.066	
Social Group Contact	136	3.9	0.4	116	3.9	0.5	0.443		54	3.8	0.8	198	3.9	0.4	0.126	
Social Experience	136	2.7	0.2	116	2.7	0.2	0.338		54	2.7	0.3	198	2.7	0.1	0.477	
Relative Social Experience Change	136	0.15	0.02	116	0.16	0.03	0.622		54	0.11	0.03	198	0.17	0.02	0.034	*
Proportion Social Experienced (%)																
Time with Juveniles (<7 years old) (%)	136	20.6	3.1	116	21.8	3.5	0.656		54	17.9	5.0	198	22.0	2.6	0.342	
Time with Mixed Sex Groups (%)	136	21.1	3.0	116	19.4	3.4	0.770		54	20.5	4.9	198	20.3	2.5	0.888	
Time Housed Separately (%)	136	24.2	2.8	116	29.4	3.4	0.616		54	29.3	5.6	198	25.8	2.4	0.948	
Time Socially Separated with Restricted Physical Access (%)	71	31.8	3.6	57	43.8	5.0	0.124		24	35.2	7.4	104	37.6	3.4	0.812	

* Indicates *P*-value<0.05

In terms of sex differences, males had a larger Overall Total Space Experience on average than females, particularly with regard to spaces that provided a choice between indoors and outdoors (Table 4). On average, males had an Overall In/Out Choice Space Experience of 25,330.7 ft^2^ compared to 13,570.1 ft^2^ for females. Additionally, females had a greater amount of Relative Social Experience Change than males (0.17 and 0.11, respectively). This indicates that while both males and females spent more time with other animals during the day than at night, females’ social group size decreased by more at night than the males’ social group size.

Comparative analysis did not detect any sex or species differences in time spent in environments with 100% coverage of hard substrates, 100% coverage of soft substrates, or 100% dirt (Table 4). Elephants spent, on average, 10.2% of their time in environments with 100% coverage of hard substrates and 10.6% in environments with 100% coverage of soft substrates. Time spent in environments with 100% coverage of either hard or soft substrate was greater during the Night than during the Day (Table 3), which indicates that elephants are more likely to be housed in single-substrate environments during the Night than during the Day.

**Fig 5:**
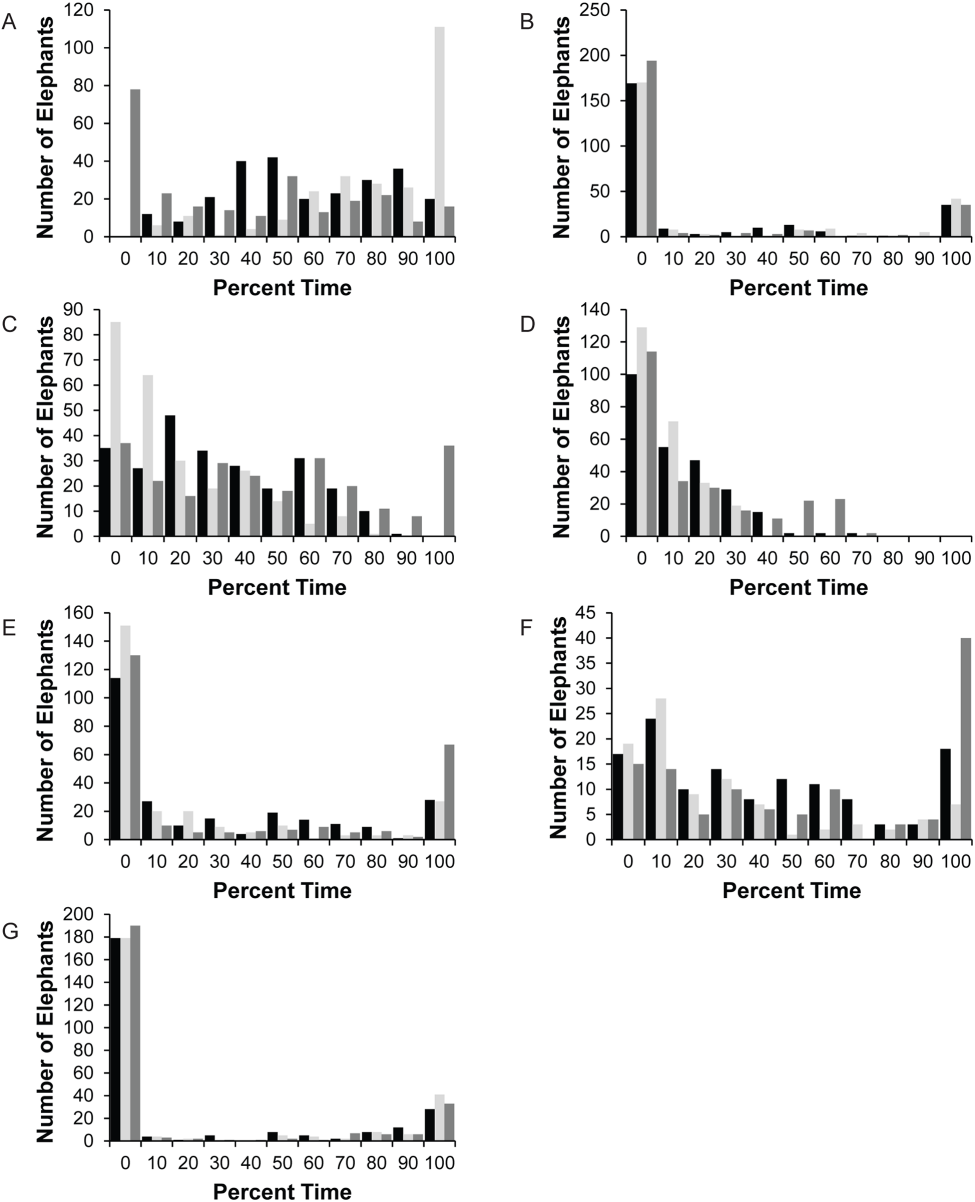
Frequency of number of elephants experiencing percentages of time for selected space and social measures. (A) Outdoors; (B) In/Out Choice; (C) Indoors; (D) On Hard Surfaces; (E) Housed Separately; (F) With Restricted Physical Access; and (G) With Juveniles (<7 years old). Bins include ranges of no experience (0%), 1–10, 11–20, 21–30, 31–40, 41–50, 51–60, 61–70, 71–80, 81–90, and 91–100% time. Dark gray bars indicate Overall experience, light gray bars indicate Daytime experience, and black bars indicate Nighttime experience.

To demonstrate the distribution of results for percentage-based variables across the population, Fig 5 presents histograms of the number of elephants that experienced between 0 and 100% time (in 10% intervals) for a number of variables. Some of these variables had bimodal distributions at the low and high ends of the scale, such as Percent Time In/Out choice, Percent Time Housed Separately, or Percent Time with Juveniles. Other measures, such as Percent Time on Hard Surfaces, showed a decrease in frequency as percent time increased. Additionally, the patterns varied between Overall and Day and Night experiences.

**Fig 6:**
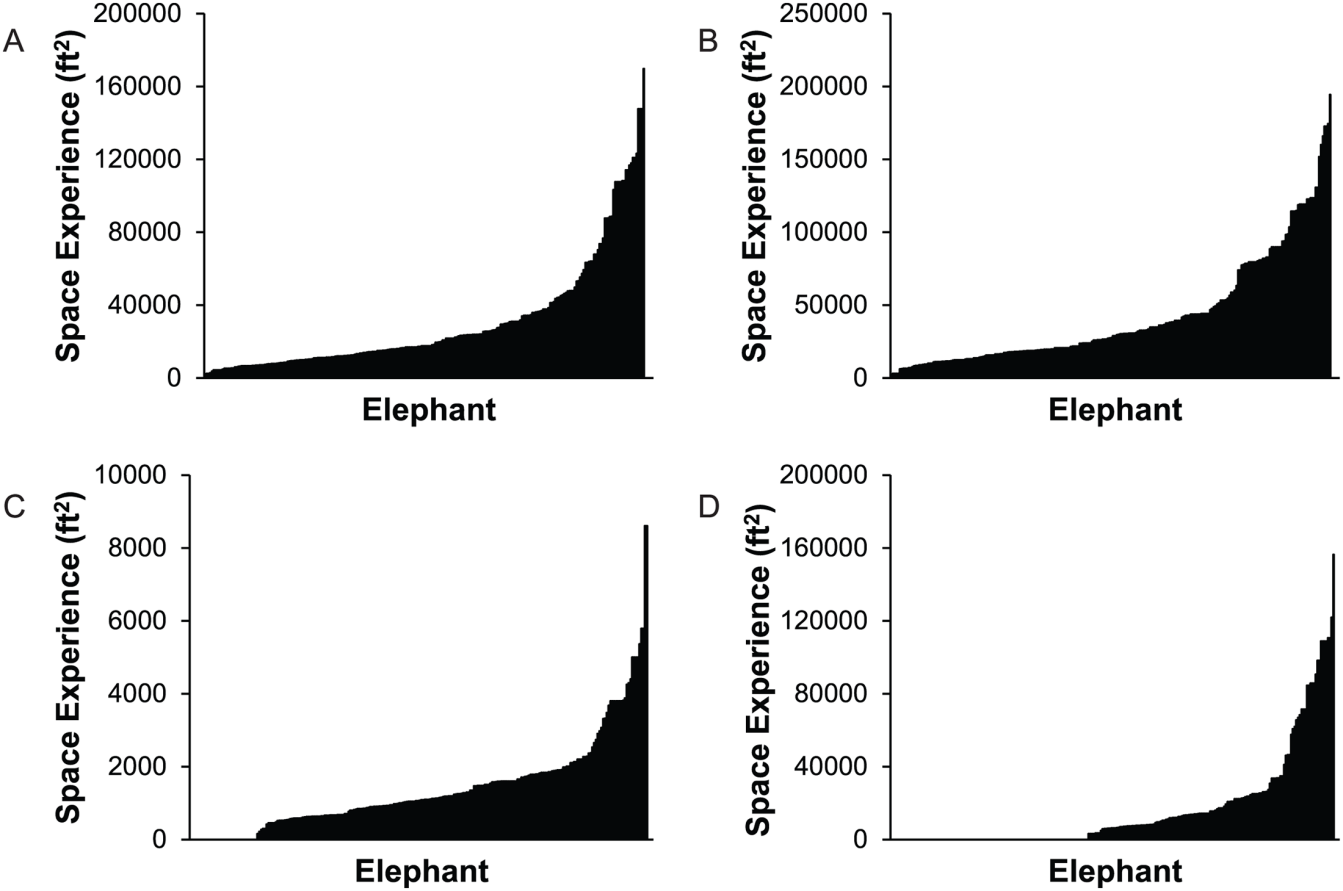
Overall Space Experience for every elephant in the study population A) Total Overall Space Experience where Space Experience is averaged over both day and night periods and includes Indoor, Outdoor, and Environments with In/Out Choice; B) Overall Space Experience Outdoor; C) Overall Space Experience Indoor; D) Overall Space Experience In/Out Choice.

To illustrate the population-level variation for the Space Experience variables, Fig 6 presents histograms illustrating each elephant’s Overall Space Experience for Indoor, Outdoor, Indoor/Outdoor Choice, and Total. It should be noted that the number of elephants represented in the Indoor and Indoor/Outdoor Choice figures are smaller than those in the Total or Outdoor figures due to the fact that some elephants spent no time in Indoor or Indoor/Outdoor Choice environments.

**Fig 7:**
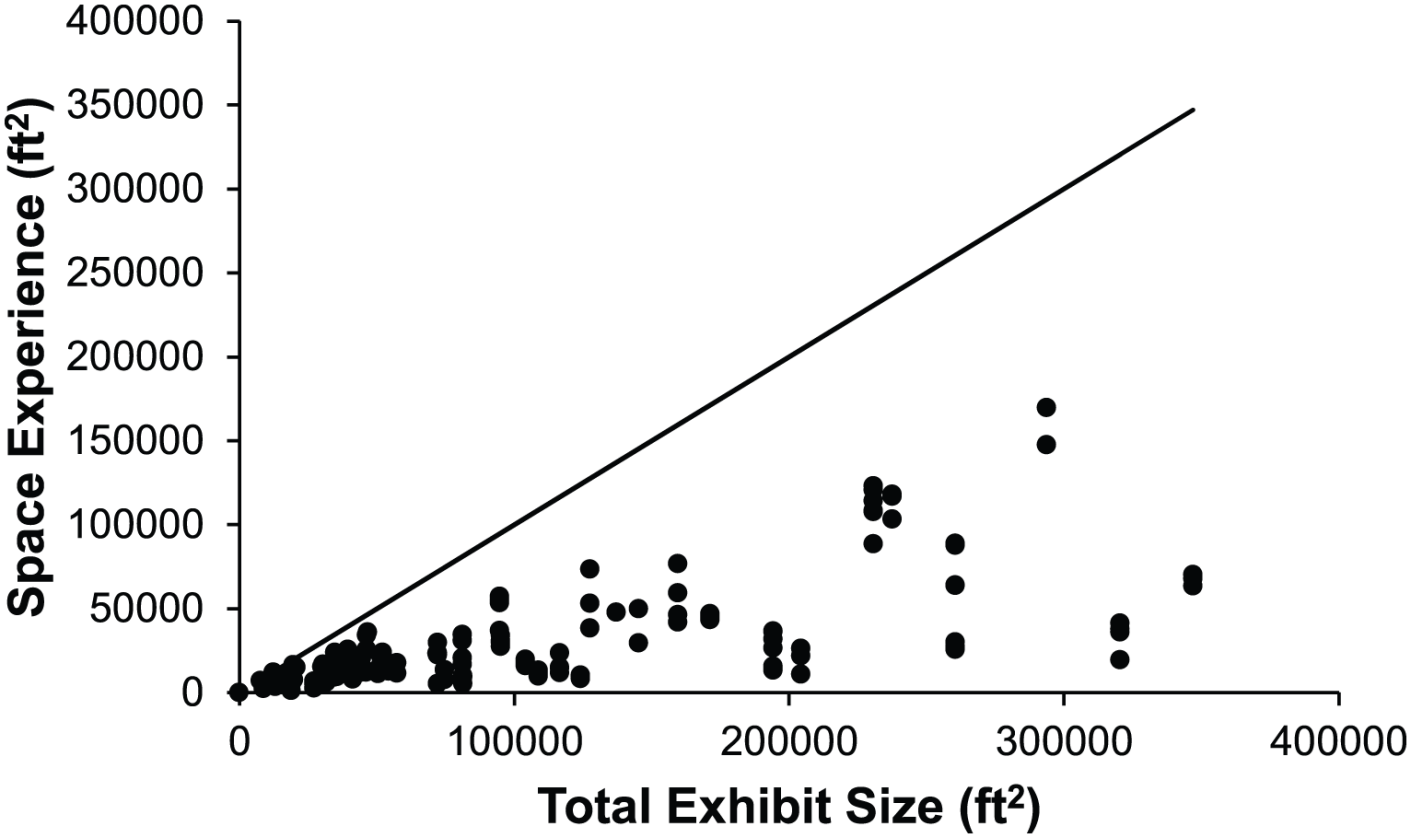
Proportion Space Experienced. Each elephant’s Overall Total Space Experience compared to the Total Exhibit Size at its zoo. The solid line represents the relationship that would exist if the elephants experienced 100% of the Total Exhibit space.

Overall Total Space Experience of an elephant did not closely correlate to the Total Exhibit Size of the zoo in which it was housed. The Wilcoxon Signed Rank test found that these two metrics of space generated significantly different (*P*-value<0.001) rankings. This difference can be readily explained by examining Proportion Space Experienced, as shown in Fig 7. The Overall Total Space Experience represented 75% or more of the Total Exhibit Size for only 11 elephants from 5 zoos, and two of those elephants were from the zoo with the smallest Total Exhibit Size. In contrast, the Overall Total Space Experience of 16 elephants from 11 zoos was less than 10% of the Total Exhibit Size for their respective zoos. On average, the Overall Total Space Experience to Total Exhibit Size ratio was 34.7%.

**Fig 8:**
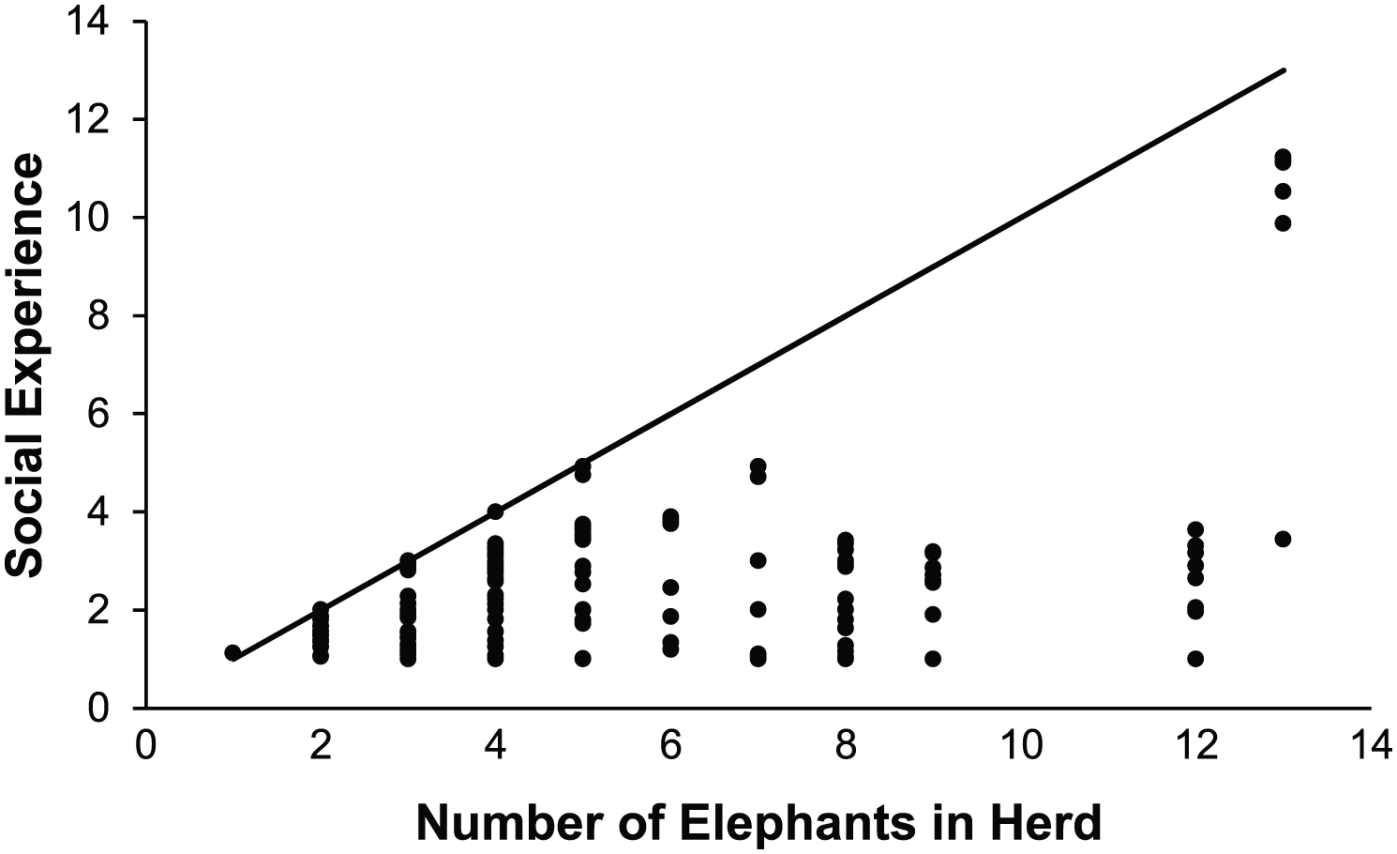
Proportion Social Experienced. Each elephant’s Overall Social Experience is compared to number of elephants in the Herd at its zoo. The solid line represents the relationship that would exist if the elephants spent all their time as a full Herd.

Similarly, the Overall Social Experience of an elephant did not correlate with Herd Size. The Wilcoxon Signed Rank test found that these two metrics provided significantly different (*P*-value<0.001) rankings, as illustrated in Fig 8. Herd Size ranged from 1 to 13 animals and an elephant’s Overall Social Experience ranged from 1 (alone) to 11.23. On average an elephant’s Overall Social Experience consisted of 60% of the herd, and at minimum 8% of the herd. Thirty-three elephants at 15 zoos had an Overall Social Experience score equal to the Herd Size and 10 elephants at three zoos had an overall Social Experience that equaled 95% of the Herd size.

## Discussion

The results presented in this paper allow for a detailed and thorough understanding of how elephants in accredited North American zoos experienced their physical and social environments over the course of a full year. The range of variables presented reflects the complexity of elephant management and underscores the need to develop comprehensive standardized methods of translating this complexity into data suitable for use in comparative analyses and in the assessment of elephant welfare.

At its simplest, the social life of a zoo elephant can be characterized by enumerating the size of the herd at the zoo at which it lives. However, modern elephant management rarely follows a model where all the elephants at a facility spend all of their time together as a single herd. In fact, elephants are managed in social groups of varying sizes and compositions, and individual elephants can be members of multiple social groups (population range: 1–30 unique social groups per elephant) with which they spend varying amounts of time depending on management schedules, elephant characteristics, or other factors. Given this approach to social management, it becomes clear that the simplest social variable, herd size, is not necessarily adequate to explain the social life of a specific individual. Animal Contact, the next simplest variable we created, improves upon herd size in that it transitions to using the individual elephant as the unit of analysis, but provides only a count of the unique elephants with which that individual shares unrestricted space. Social Group Contact is a more complex variable that is also calculated at the elephant level, but in addition incorporates the factor of groupings to account for the fact that elephants experience diversity in their social group membership. Finally, Social Experience accounts for the factor of time in elephant social management by weighing the size of each social group experienced by the amount of time each elephant spends in each group. The fact that Social Experience integrates the number of elephants in each social grouping with the element of time makes it the most robust approach to standardizing the quantification of complex managed social milieus developed to date.

In addition to quantifying an elephant’s social experience with respect to number of conspecifics, we calculated additional social variables such as percent time housed with juveniles, in a mixed sex group, separately, or separately with restricted physical access to conspecifics. Wild elephant herds typically include calves and juveniles. Allomothering,which is the caretaking of the offspring of herdmates, is prevalent and believed to provide pre-pubescent and/or nulliparous females with valuable mothering experiences and skills [38, 39]. Thus, the presence of calves and juveniles within a captive herd may have important ramifications for the successful rearing of offspring for first-time mothers. In addition, in many species juveniles are known to engage in more play than adults [40], and their presence in an elephant herd is hypothesized to add to the dynamic nature of group interactions in a way that supports normal behavioral expression [9]. In our population, 45 of the 226 adult elephants (36/181 females and 9/45 males) had the opportunity to spend time with juveniles. The mean time spent with juveniles by these elephants was 65.68%. Thus, while about two-thirds of the social time budget of these elephants was spent in social groupings that included juveniles, the opportunity for social interaction with young elephants was only available to 20% of the adult elephants in the population.

Quantifying an elephant’s physical environment with respect to space requires standardization of complex management models that vary both within and between zoological settings. Elephant management rarely involves the housing of elephants in a single defined area, but rather involves shifting individuals or groups between a variety of spaces of differing sizes and features. In fact, elephants in the study population spent time in an average of 9.4 different environments in the course of regular management (population range: 2–46). To account for this practice, we looked at space allocation using multiple variables, ranging from the simplest zoo-level variable (Total Exhibit Size) to the most complex variable that accounted for different amounts of time spent in environments of differing sizes (Space Experience). In addition, housing variables were calculated to quantify both Space Experience and Percent Time in different types of environments (Indoor, Outdoor, In/Out Choice) and for different time periods (Day, Night, Overall). This yielded a variety of specific variables, each of which characterized space by accounting for relevant characteristics of the physical environment. Knowledge of these aspects of elephant housing are important given that, in other species, positive associations have been found between improvements in behavioral and/or physiological indicators of welfare and housing animals outdoors[41, 42, 43,44] or in environments that provide indoor/outdoor choice [45,46,47,48].

The Proportion Space and Social Experienced analysis was conducted to reveal how each individual elephant’s experience of physical and social resources correlated with the total resources available at the zoo. Mean proportion experienced was 34.7% for spatial resources (Fig 7). While we do not know if it is logistically possible to achieve Space Experience scores that are 100% of Total Exhibit size (due to the layout of each exhibit), it is clear that there is opportunity to more efficiently utilize spatial resources by offering access to multiple contiguous enclosures at the same time. For social resources, the mean proportion experienced was 60%. While there could be many factors contributing to spatial and social resources not being fully utilized, our data indicate that these rates are being driven mainly by practices associated with Night management. The Relative Space Experience Change and Relative Social Experience Change variables (Fig 4) demonstrate that 199/252 elephants in the population had a restriction in Space Experience and 148/252 had a restriction in Social Experience when moving from Day management to Night management. Across the population, Relative Change in Social Experience represents a decrease of 1.2 animals and the Relative Change in Space Experience represents a decrease of 22897.2 ft^2^ from Day to Night. This trend in management is particularly notable given the fact that the Night management period was reported as ranging from 8–18 hours depending on the season with a modal value of 14 hours in both the summer and winter (Fig 3), and given that Night Social Experience was found to be significant predictor of nighttime stereotypy performance in a related study [9].

Exposure to hard substrates has been hypothesized to be associated with the prevalence of foot and musculoskeletal problems in elephants [29] and decreases in foot health and recumbent resting behavior in cattle [28, 33, 49]. Our analysis provided a conservative estimate of time spent on hard surfaces due to the fact that we were only able to capture time spent in environments with 100% coverage of either hard or soft substrates. However, the variability in substrate exposure across the population was sufficient to test for associations between exposure to hard or soft substrates and behavioral and health-related indicators of welfare in related studies [10, 11,13].

While the study population as a whole displayed a wide range of variation in many of the housing and social variables, only a few significant differences were found between the two species (Asian / African) and by sex, indicating that dissimilarities in elephant management practice do not occur consistently along species or sex lines. For the species comparisons, the most notable differences were in Space Experience. The mean Overall Total Space Experience for African elephants was 39,338.8 ft^2^, which is more than twice that of Asian elephants where the mean value was 19,264.2 ft^2^. The data suggest that the difference in Space Experience between the two species is driven by differences in the area of outdoor space available in exhibits, as Overall Outdoor Space Experience was also significantly greater for African elephants. Space Experience also diverged between the sexes. Both Overall Total Space Experience and Overall In/Out Choice Space Experience were significantly higher for males. The management of male elephants generally requires larger and more flexible housing resources; and these results indicate that these resources are being used such that males spend more time in larger, outdoor environments than females.

Our analyses demonstrated that the zoo-level factors Herd Size and Total Exhibit Size were not correlated with the individual-level Social Experience or Space Experience scores. This indicates that, because of the complex ways in which elephants are managed, zoo level factors are not a proxy for individual elephant experience. This is a particularly relevant finding to animal welfare assessment, because welfare outcomes such as behavior and physiology are sensitive to differences in physical and social milieu and associations could be masked if zoo-level, rather than individual-level independent variables are used. In fact, when variables presented in this paper were tested in multi-variable predictive models for a range of welfare indicators, those that were calculated at the zoo or herd level were never significant factors, whereas many of the individual elephant-level variables were significantly associated with elephant welfare outcomes (see: [9,10,11,12,13,14]).

## Conclusion

Resource based measures that describe housing and management practices are necessary to provide a comprehensive assessment of the welfare of managed animals. We have described the development of such variables created as part of an epidemiological study assessing the welfare of elephants in North American zoos [50]. We found that although there was variability in how elephants are housed in terms of space, flooring, and social groups, these differences were rarely associated with the species or sex of the elephants. In addition, for both spatial and social measures, individual and zoo based variables were not correlated. These results, combined with the finding that Day and Night management varied with respect to key housing and social factors, highlight the need for individual-based variables that represent both operating and non-operating zoo hours to adequately represent animals’ experiences. These can then be utilized as standardized variables for assessing resource based measures and their associations with welfare indicators both within and across institutions. This approach may be applicable to any zoo-housed animal, but is particularly relevant to those species that are managed dynamically.

## Supporting Information

S1 Table Metric equivalents for Space Experience variables. (XLSX)
